# Categorizing metadata to help mobilize computable biomedical knowledge

**DOI:** 10.1002/lrh2.10271

**Published:** 2021-05-09

**Authors:** Brian S. Alper, Allen Flynn, Bruce E. Bray, Marisa L. Conte, Christina Eldredge, Sigfried Gold, Robert A. Greenes, Peter Haug, Kim Jacoby, Gunes Koru, James McClay, Marc L. Sainvil, Davide Sottara, Mark Tuttle, Shyam Visweswaran, Robin Ann Yurk

**Affiliations:** ^1^ Computable Publishing LLC Ipswich Massachusetts USA; ^2^ Medical School University of Michigan Ann Arbor Michigan USA; ^3^ Biomedical Informatics and Cardiovascular Medicine School of Medicine, University of Utah Salt Lake City Utah USA; ^4^ Taubman Health Sciences Library, University of Michigan Ann Arbor Michigan USA; ^5^ School of Information University of South Florida Tampa Florida USA; ^6^ College of Information Studies University of Maryland College Park Maryland USA; ^7^ Arizona State University and Mayo Clinic. Scottsdale Arizona USA; ^8^ Intermountain Healthcare University of Utah Salt Lake City Utah USA; ^9^ Komodo Health San Francisco California USA; ^10^ Department of Information Systems University of Maryland Baltimore Maryland USA; ^11^ Emergency Medicine University of Nebraska Medical Center Omaha Nebraska USA; ^12^ Mayo Clinic Scottsdale Arizona USA; ^13^ Apelon Hartford Connecticut USA; ^14^ Department of Biomedical Informatics University of Pittsburgh Pittsburgh Pennsylvania USA; ^15^ MDyurk West Bloomfield Michigan USA

**Keywords:** computable biomedical knowledge, digital objects, FAIR principles, metadata, trust

## Abstract

**Introduction:**

Computable biomedical knowledge artifacts (CBKs) are digital objects conveying biomedical knowledge in machine‐interpretable structures. As more CBKs are produced and their complexity increases, the value obtained from sharing CBKs grows. Mobilizing CBKs and sharing them widely can only be achieved if the CBKs are findable, accessible, interoperable, reusable, and trustable (FAIR+T). To help mobilize CBKs, we describe our efforts to outline metadata categories to make CBKs FAIR+T.

**Methods:**

We examined the literature regarding metadata with the potential to make digital artifacts FAIR+T. We also examined metadata available online today for actual CBKs of 12 different types. With iterative refinement, we came to a consensus on key categories of metadata that, when taken together, can make CBKs FAIR+T. We use subject‐predicate‐object triples to more clearly differentiate metadata categories.

**Results:**

We defined 13 categories of CBK metadata most relevant to making CBKs FAIR+T. Eleven of these categories (type, domain, purpose, identification, location, CBK‐to‐CBK relationships, technical, authorization and rights management, provenance, evidential basis, and evidence from use metadata) are evident today where CBKs are stored online. Two additional categories (preservation and integrity metadata) were not evident in our examples. We provide a research agenda to guide further study and development of these and other metadata categories.

**Conclusion:**

A wide variety of metadata elements in various categories is needed to make CBKs FAIR+T. More work is needed to develop a common framework for CBK metadata that can make CBKs FAIR+T for all stakeholders.

## 1 INTRODUCTION

Computable biomedical knowledge artifacts (CBKs) are digital objects carrying biomedical knowledge represented in data structures that can be parsed and processed by a machine.^1^, ^2^, ^3^ The range of content represented in CBKs spans all biomedical knowledge, including knowledge about atoms, molecules, cells, organs, individual people, human populations, and the environments in which people live. The creation of CBKs is widespread, but it is currently difficult to find, apply, and use CBKs broadly. The purpose of this article is to provide an outline that scopes a future CBK metadata framework to help mobilize CBKs by making them findable, accessible, interoperable, reusable, and trustable (FAIR+T).^4^, ^5^

### 1.1 CBKs are variable and important

CBKs vary in their content, purpose, and audience. Some CBKs support biomedical research or population health analytics. Others help improve health outcomes by enabling clinical decision support, health education, health promotion, or behavior change. In some instances, CBKs have multiple uses that span research, education, clinical care, population health, and public health.

Different types of CBKs exist, including bibliographic records,^6^, ^7^ value sets,^8^ terminologies and ontologies,^9^, ^10^ computable phenotypes,^11^ computable recommendations from guidelines,^12^ computable evidence resources, predictive models,^14^ causal models,^15^ and business process and workflow models.^16^

Many people publish CBKs so they can be replicated, reproduced, and used by others.^17^ CBKs produced by data scientists and knowledge engineers are an increasingly common form of scholarly communication.^18^ Following the example set by journals in computer science, biomedical journals are beginning to support CBK publication.^19^

CBKs are essential for large‐scale initiatives such as *precision health* ^20^ and *learning health systems*.^21^ Achieving the Quintuple Aim (a framework for the comprehensive approach to defining healthcare quality with five broad outcomes of lowering cost, improving population health, optimizing patient experience, assuring care team well‐being, and ensuring equity and inclusion)^22^ will require a systematic application of complex CBKs on a massive scale.

Students and clinical educators are also CBK stakeholders. As curricula throughout biomedicine evolve, we anticipate more students will develop and use CBKs during their training for careers in biomedical science, the health professions, and related disciplines.^23^

As CBKs become more numerous, powerful, and complex, the value of structured, searchable metadata grows for producers to share their CBKs, curators to organize CBKs, and consumers to find, deploy, and use CBKs more easily. This article outlines categories of metadata for describing CBKs sufficiently to enable CBKs to be widely shared and mobilized for their various purposes. We focused specifically on CBK metadata categories that can make CBKs FAIR+T.

## 2 BACKGROUND AND SIGNIFICANCE

### 2.1 Functional view of CBKs

All CBKs are digital objects (DOs). Work on metadata for DOs predates Kahn and Wilensky's 1995 work on distributed digital object services.^24^ Three key components of all DOs are content (in the form of a bit sequence), a unique identifier, and describable properties (eg, size in bits).^4^, ^24^, ^25^

CBKs are often custom‐built and incorporated into larger software applications in ways that make them difficult to identify, isolate, extract, and share.^26^ However, we assume that all CBKs can be isolated and shared as independent DOs, depending on software design.^27^, ^28^ We further assume that isolating CBKs is a precursor to mobilizing them. Therefore, we do not consider applications (apps) or software services (APIs) that incorporate CBKs to be CBKs themselves. Instead, we view CBKs as the smaller DO components of apps and APIs that represent biomedical knowledge in concrete, machine‐independent encodings or data structures.^4^, ^27^ CBKs may either be standalone or be embedded within apps, APIs, information systems, or platforms.

We draw on multiple perspectives about different CBK types. First, CBK types may reflect the structured machine‐interpretable formats or languages used to represent their knowledge content (eg, JSON, propositional logic, or Python).^29^ Second, CBKs may be distinguished by their place in a hierarchy of increasing CBK complexity, such as building on basic CBKs like terms and relationships and constructing increasingly complex composite CBKs such as decision trees, workflows, and plans.^29^, ^30^ Third, it is clear from real‐world examples that CBKs may also be typed according to their logic or purpose (eg, rule, predictive model, risk‐scoring mechanism). To demonstrate and contextualize our ideas about different CBK types, we provide 12 examples of CBKs in our supplement (see Supplement).

In summary, we view CBKs as DOs that are concrete, distinct, shareable *information content entities*.^31^, ^32^ Some CBKs represent and communicate knowledge as assertions with an evidential basis. In general, CBKs explicitly represent and convey biomedical knowledge that holds significance for an identified community.^1^, ^33^ Their explicitness enables CBKs to be immediately processed or executed by digital computers. Because CBKs are increasingly important throughout biomedicine, there is a vast and diverse audience for this work to help mobilize CBKs. Mobilizing CBKs means making them available wherever they can be appropriately used to advance biomedical science and improve human health.

### 2.2 Mobilizing CBKs as strategy to add value and increase impact

The members of the Mobilizing Computable Biomedical Knowledge (MCBK) Community (www.mobilizecbk.org) call for the development of open, safe, effective, equitable, and inclusive CBKs that are FAIR+T.^34^ The MCBK Community has four workgroups. The authors of this article are all volunteer members of the MCBK Community's Standards Workgroup. As part of this effort, we periodically engaged the broader Standards Workgroup and MCBK Community to obtain feedback, but the authors are solely accountable for the contents of this article.

To assist specifically with CBK findability and access, repositories for CBKs are emerging. Two examples of public CBK repositories are CDS Connect^35^ and the Value Set Authority Center.^36^ Other examples include the computable phenotype repository PheKB,^11^ the Kipoi repository of predictive models for genomics,^14^ and the DDMORE repository of computable models for pharmaceutics.^37^ Some suggest that private software code repositories, such as GitHub, Source Forge, and Bitbucket, are suitable for hosting CBKs.^38^ However, others point out the policies governing these repositories may not fully support the CBK long‐term sharing needs of biomedical scientists.^39^, ^40^ We assume, in the future, there will be many CBK repositories and CBK metadata registries supporting a robust CBK ecosystem.

### 2.3 Using metadata as a strategy to mobilize CBKs

There exist extensive prior bodies of work on metadata, for example, those described in Greenberg's 2017 overview entitled, “Metadata and Digital Information.^41^” Since the 1960s, metadata developments within and beyond the digital library community have significantly matured.^41^ It is clear that different communities value metadata for different reasons, such as the library community emphasizing descriptive metadata for distinguishing information resources and the business community emphasizing machine processing of metadata to improve information systems. The purpose of this manuscript is to highlight categories of metadata to assist in greater sharing and dissemination of CBKs. We are not attempting here to provide a comprehensive framework for metadata formalism or to create a standard, such as ISO/IEC 11179‐3:2013 which specifies the structure of a metadata registry in the form of a conceptual data model.

It is clear specific metadata can support CBK sharing and use.^42^ Much prior work focuses on making *data sets* FAIR.^4^ Organizations and efforts like FORCE11,^42^ CEDAR,^43^, ^44^ GO FAIR,^45^ DataCite,^46^ and the Research Data Alliance^47^ are advancing support for metadata about scientific data sets. We build on existing efforts to enhance data set metadata to develop metadata categories to make CBKs FAIR+T.

We anticipate that the production of CBKs will continue to increase as it has since the 1970s.^48^ Mobilizing the growing number of CBKs for optimal use requires them to be well organized and managed. This work significantly advances a metadata strategy to mobilize CBKs. Just as other classes of digital artifacts (eg, music and video files) have been mobilized in part by using rich metadata, further development of metadata for CBKs should enable them to be widely shared and appropriately used for research, education, health promotion, health care, population health, and public health. Outlining the metadata that can make CBKs FAIR+T is an initial step in a larger mobilization strategy.

Our goal is to engage both the many who have previously advanced our theory and practice in metadata usage and the many who are currently developing applications within specific domains, to facilitate development of a CBK metadata framework to help mobilize CBKs across a wide spectrum.

There are several unique aspects (individually or in combination) to our current effort. First, our focus is on specification of metadata for *computable* knowledge artifacts. Second, our description of metadata elements includes subject‐predicate‐object triples to enable clear definitions and reduce overlaps across metadata categories. Third, although we do not presume any specific application of our metadata categories, we are approaching this work with a primary focus of functional application and thus limiting attention to metadata that is mainly for a FAIR+T purpose. Even so, our current approach is purely conceptual and independent of any particular application and/or realization of the metadata, so it could be easily adapted in subsequent efforts to provide a reference framework for both existing and future implementations for a common meaning and purpose, enabling interoperability in the process. In particular for repositories, we envision ecosystems where the metadata records themselves are implemented as CBKs.

## 3 RESEARCH QUESTION

What categories of metadata hold the potential to make CBKs findable, accessible, interoperable, reusable, and trustable (FAIR+T)?

## 4 METHODS

Our group of researchers, data scientists, knowledge engineers, and clinicians collaborated to develop and describe a list of CBK metadata categories. Our overarching goal was to determine which categories of metadata may play a significant role in making CBKs FAIR+T.

Regular weekly videoconferences and other small group meetings throughout the calendar year 2020 enabled us to coordinate and advance our work. Five phases of group effort led to the development of our final CBK metadata category list: (1) performing an environmental scan, (2) surfacing candidate metadata categories, (3) deciding upon an initial CBK metadata category list, (4) gathering feedback from the wider MCBK community on an initial draft categories list, and (5) resolving inconsistencies and overlap to arrive at a final metadata categories list.

### 4.1 Phase 1—Environmental scan

We conducted a rapid environmental scan to identify key types of metadata specified in existing standards, for example, Dublin Core. In addition, this scan surfaced real‐world examples of existing metadata describing actual CBKs in online repositories. Overall, we reviewed metadata and metadata categories from Health Level 7 International (HL7), Dublin Core, Schema.org, Object Management Group (OMG.org), GitHub, The Future of Research Communication and e‐Scholarship (FORCE11), and the Library of Congress. Next, we compiled information about specific metadata elements, types of metadata, and categories of metadata into a shared spreadsheet.

### 4.2 Phase 2—Surfacing candidate metadata categories

During the spring of 2020, we iteratively analyzed potential metadata categories by applying an evolving list of categories to a *convenience sample* of several real‐world CBKs (see Supplement). Our example CBKs were all accessible online and came with metadata from their existing repositories.

For each candidate metadata category, we listed specific metadata elements from the category. Next, we attempted to identify prior published works about each candidate metadata category in our list. During this phase, we also explored how metadata elements in each candidate category assist in making CBKs FAIR+T.

After several cycles of applying our CBK metadata categories list to these actual CBK examples, discussing the categories list and the CBK examples together, and refining our categories list further, we realized 15 candidate metadata categories for an initial draft of our CBK metadata list.

### 4.3 Phase 3—Deciding upon an initial metadata categories list

When deciding on which metadata categories to keep and which to combine or set aside, we gave preference to previously defined metadata categories over new categories. As part of our decision‐making process, we clarified the scope of the metadata categories in our initial list by collaboratively drafting and revising a paragraph outlining each category's scope. We agreed upon a list of 11 metadata categories at this intermediate stage.

### 4.4 Phase 4—Collecting and responding to feedback from the wider MCBK community

In advance of the MCBK Community's Annual Meeting at the end of June and the beginning of July 2020, we produced a draft document describing our initial metadata categories. This draft document conveyed our initial metadata categories list and described each category in detail. At the Annual Meeting, we convened the MCBK Community's Standards Workgroup and gathered feedback on our preliminary metadata categories list. We organized breakout sessions to discuss four metadata categories in particular (Biomedical Domain, Coverage, Purpose, and Type).

After the MCBK Community's Annual Meeting in 2020, we consolidated our meeting notes and the feedback we obtained from Standards Workgroup members about our preliminary metadata categories into a summary document. We circulated that summary document throughout our group of authors and discussed the feedback we received in detail. As a result, an updated but still unfinished list of metadata categories emerged by the end of August 2020.

### 4.5 Phase 5—Removing inconsistencies and overlap to arrive at a final metadata categories list

We created our final list of CBK metadata categories using an iterative process. During this process, to address overlap, we developed and repeatedly applied a method of specifying subject‐predicate‐object triples for each metadata category. Making these triples explicit provided us with a needed mechanism to see, discuss, and address several significant problems of category overlap.

Finally, we further clarified the scope of the metadata categories in our working list by drafting and revising a paragraph outlining each category's scope. Once our group decided upon a set of metadata categories for our final list, we examined and discussed the final list to generate a related CBK metadata research agenda focused on remaining issues and areas of ambiguity. This research agenda describes future work toward having sufficient metadata to make CBKs FAIR+T.

## 5 RESULTS

### 5.1 List and description of metadata categories

We generated a final list of 13 categories of CBK metadata elements with specific utility for making CBKs FAIR+T. In Table 1, we classify each category according to the principle to which it most closely applies. We briefly summarize the elements included in each category, offer some example predicates, and complete Table 1 with references for precedents in each metadata category. The text provides a more detailed narrative description of each category with examples drawn from actual CBKs.

**TABLE 1:** List of metadata categories related to making CBKs and FAIR+T

Metadata category	Metadata elements in this category	Example predicates	Main principle supported	From
1. Type	Elements that classify CBKs by describing the **nature** of CBKs in some general way	[CBK] *is_a* {type}	FINDABLE	49, 50
2. Domain	Elements relating CBKs to the **biomedical domains or topics** to which they belong	[CBK] *is_about* {domain}	FINDABLE	51, 52
3. Purpose	Elements describing the **purposes** or circumscribing and limiting the **intended uses** of CBKs	[CBK] *has_purpose_of* ____	FINDABLE	53
		[CBK] *is_intended_to* ____		
		[CBK] *is_not_intended_to* ____		
4. Identification	Elements indicating **persistent identifiers** or **persistent unique identifiers** and **versions** assigned to CBKs	[CBK] *has_identifier* ____	FINDABLE	49, 50
		[CBK] *has_name* ____		
		[CBK] *has_version* ____		
5. Location	Elements indicating the **physical or virtual locations** where CBKs can be accessed	[CBK] *has_location* {ADDRESS}	ACCESSIBLE	49, 50
		[CBK] *is_located_at* {URL}		
6. CBK‐to‐CBK relationships	Elements describing a **relationship between one CBK and some other CBK**	[CBK] *is_modification_of* [CBK]	INTEROPERABLE	49, 50
		[CBK] *is_predecessor_of* [CBK]		
		[CBK] *is_successor_of* [CBK]		
		[CBK] *is_used_with* [CBK]		
7. Technical	Elements to describe a wide array of **technical characteristics** of CBKs that need to be known to deploy, integrate, operate, and use them	[CBK] *has_file_type* ____	INTEROPERABLE	54, 55
		[CBK] *has_file_size* ____		
		[CBK] *has dependency* ____		
		[CBK] *can be executed using* ____		
		[CBK] *has input* ____		
		[CBK] *has output* ____		
8. Authorization and rights management	Elements describing **rights and responsibilities** pertaining to CBKs	[CBK] *is_available_to* [person]	REUSABLE	56
		[CBK] *has_license* [license]		
		[CBK] *copyright_held_by* [agent]		
		[CBK] *has_disclaimer* [disclaimer]		
9. Preservation	Elements needed to **archive** CBKs for decades‐ long periods of time with minimal degradation	[CBK] *has_preservation_level* [level]	REUSABLE	57
		[CBK] *should_be_kept_until* [date]		
10. Integrity	Elements conveying **outputs from cryptographic functions** that allow CBK users to confirm CBK has not been tampered with	[CBK] *has_hash* [hash function output]	REUSABLE	58
		[CBK] *uses_hash_function_type* [type]		
11. Provenance	Elements indicating **changes in ownership**, **custody, and status** during CBK lifecycles	[CBK] *is_owned_by* [agent]	TRUSTABLE	59
		[CBK] *ownership_changed_on* [date]		
		[CBK] *has status* [status]		
		[CBK] *status_changed_on* [date]		
		[CBK] *is_authored_by* [author]		
		[CBK] *is_reviewed_by* [reviewer]		
		[CBK] *is_endorsed_by* [endorser]		
*Two evidence categories*				
12. Evidential basis	Elements describing the **data upon which the claims in CBKs are based**, the **methods of obtaining and analyzing those data**, and the **strength** of the evidential basis of CBKs.	[CBK] *is_based_on_data_about* ____	TRUSTABLE	2, 60, 61, 62
		[CBK] *is_based_on_data_colleted_at* [place]		
		[CBK] *is_based_on_data_collected_by* [agent]		
		[CBK] *is_based_on_data_collected_on* [date]		
		[CBK] *is_based_on_data_collected_for* ____		
		[CBK] *is_based_on_data_analysis_method_of*		
		[CBK] *is_based_on_data_analysis_results_of*		
		[CBK] *has_certainty_of_evidence* ____		
13. Evidence from use	Elements describing **data arising from CBK use,** the **methods of obtaining and analyzing those data**, and the **strength** of evidence about CBK use	CBK] *use_is_evaluated_in* ____	TRUSTABLE	61, 62, 63
		[CBK] *use_is_associated_with* ____		
		[CBK] *use causes ____*		
		[CBK] *use_evidence_has_certainty_of ____*		

To provide illustrative examples, we show CBK metadata for 12 examples of actual CBKs used for clinical decision support, biomedical research, or population and public health. Details about these CBK examples are listed next in Table 2. The first four CBK examples, referred to by the capital letters A‐D, are referenced repeatedly in the descriptions of metadata categories below. A series of more highly elaborated examples of actual CBKs, each with a panel of metadata reflecting many of the 13 metadata categories in Table 1, appears in the Supplement.

**TABLE 2:** Actual computable biomedical knowledge artifacts (CBK) used as examples for results

CBK example	CBK citation	CBK description
A	Statin Use for the Primary Prevention of CVD in Adults: Patient‐Facing CDS Intervention [Clinical Decision Support Artifact], version 0.1. Contributors: The MITRE Corporation, US Preventive Services Task Force [Contributors], Agency for Healthcare Research and Quality [Steward]. In: CDS Connect. Created June 1, 2019. Approved September 8, 2019. Accessed December 5, 2020. Available at: https://cds.ahrq.gov/cdsconnect/artifact/statin‐use‐primary‐prevention‐cvd‐adults‐patient‐facing‐cds‐intervention.	A clinical decision support artifact of subtype Event‐Condition‐Action Rule that supports presenting recommendations for use of statins in response to patient characteristics representing increased risk for cardiovascular disease.
B	SeqVec/embedding2structure [Model]. Contributor: Michael Heinzinger [Author]. In: Kipoi.org, doi 10.1101/614313. Accessed December 5, 2020. Available at: http://kipoi.org/models/SeqVec/embedding2structure/. Computable resource at: https://github.com/kipoi/models/tree/master/SeqVec/embedding2structure.	A dataset for a prediction model for a three‐state, eight‐state secondary structure and disorder prediction based on SeqVec.
C	Innate Inflammation; model 2018 [Model], version 1. Contributors: Hans Westerhoff [Contributor, Submitter], Ablikim Abudukelimu [Contributor]. In: FAIRDOM Hub, model 640. Created November 5, 2019. Accessed December 6, 2020. Available at: https://fairdomhub.org/models/640. Computable resource at: https://fairdomhub.org/models/640/download?version=1.	A model of type ordinary differential equations used with Copasi to obtain the figures of Abudulikemu 2018 Predictable Irreversible Switching Between Acute and Chronic Inflammation.
D	Anthrax Post‐Exposure Prophylaxis [Clinical Decision Support Artifact], version 0.2. Contributors: The MITRE Corporation [Contributor], Centers for Disease Control and Prevention [Steward]. In: CDS Connect. Created October 25, 2018. Approved August 6, 2020. Accessed December 5, 2020. Available at: https://cds.ahrq.gov/cdsconnect/artifact/anthrax‐post‐exposure‐prophylaxis.	A clinical decision support artifact of subtype multimodal that supports presenting recommendations for evaluation and management of adults exposed to anthrax within the past 60 days.
E	Calculator: Cardiovascular risk assessment in adults (10‐year, ACC/AHA 2013) (Patient education) [Interactive Form], version 3.0. In: EBMcalc in UpToDate, Topic 119 179. Accessed December 5, 2020. Available at: https://www.uptodate.com/contents/calculator‐cardiovascular‐risk‐assessment‐in‐adults‐10‐year‐acc‐aha‐2013‐patient‐education.	An interactive calculator to receive input of patient characteristics and provide an output of a predicted risk for cardiovascular events within 10 years.
F	CHA₂DS₂‐VASc Score for Atrial Fibrillation Stroke Risk [Interactive Form]. Contributors: Calvin Hwang [Content Contributor], Gregory Lip [Creator of risk score]. In: MDCalc platform. Created September 17, 2009. Accessed March 14, 2021. Available at: https://www.mdcalc.com/cha2ds2‐vasc‐score‐atrial‐fibrillation‐stroke‐risk.	An interactive calculator to receive input of patient characteristics and provide an output of a predicted risk for stroke related to atrial fibrillation.
G	Diabetes [Terminology], version 20 190 315. Contributors: National Committee for Quality Assurance [Steward]. In: Value Set Authority Center, OID 2.16.840.1.113883.3.464.1003.103.12.1001. Accessed October 27, 2020. Available at: https://vsac.nlm.nih.gov/valueset/2.16.840.1.113883.3.464.1003.103.12.1001/expansion/Latest [Login required]. Computable resource with: API or Excel export.	A set of values (terminology codes) for the condition of diabetes.
H	Electronic Health Record‐based Phenotyping Algorithm for Familial Hypercholesterolemia [PseudoCode], version 2.0. Contributors: Iftikhar Kullo [Principal Investigator, Author], Adelaide Arruda‐Olson, Carin Smith, Hongfang Liu, Majid Rastegar, Maya Safarova, Parvathi Balachandran, Saeed Mehrabi, Sunghwan Sohn, Xiao Fan, Yijing Cheng [Authors]. In: Phenotype Knowledgebase (PheKB). Created June 2016. Accessed May 12, 2020. Available at: https://phekb.org/sites/phenotype/files/FH_eAlgorithm_Pseudocode_FullText_2016_1_3.pdf.	A pseudocode expression of a computable phenotype to classify people as cases or controls for familial hypercholesterolemia based on data in the electronic health record.
I	Antibiotic Resistance Ontology (ARO) [Terminology], version 1.0. In: OBO Library, entry aro. Revised August 2020. Accessed December 6, 2020. Available at: https://github.com/arpcard/aro. Computable resource at: https://raw.githubusercontent.com/arpcard/aro/master/aro.owl.	An ontology related to antibiotic resistance.
J	Endocrinology: Hypoglycemia Order Set [Clinical Decision Support Artifact], version 1.0. Contributors: Leonard Pogach, Paul Conlin [Contributors], Veterans Health Administration [Steward]. In: CDS Connect. Created April 20, 2018. Approved March 25, 2019. Accessed May 12, 2020. Available at: https://cds.ahrq.gov/cdsconnect/artifact/endocrinology‐hypoglycemia‐order‐set.	A clinical decision support artifact of subtype order set that facilitates next steps in response to occurrence of a hypoglycemic event, or presence of risk factors for hypoglycemia, by presenting orders for medications, supplies, laboratory tests, point of care tests, consults and referrals, and patient and caregiver education.
K	Citation for FEvIR Evidence 55 [FHIR Resource]. Contributors: Brian S Alper [Author]. In: Fast Evidence Interoperability Resources (FEvIR) Platform, entry 58. Created March 13, 2021. Accessed March 13, 2021. Computable resource at: https://fevir.net/resources/Citation/58.	A Fast Healthcare Interoperability Resources (FHIR) Resource of type Citation which provides the citation information for FEvIR Resource 55 of type Evidence.
L	14‐day mortality Remdesivir vs placebo meta‐analysis (ACTT‐1, Wang et al, WHO SOLIDARITY) [FHIR Resource], version 4. Contributors: Brian S Alper, Joanne Dehnbostel, Khalid Shahin [Authors]. In: Fast Evidence Interoperability Resources (FEvIR) Platform, entry 55. Created December 17, 2020. Revised December 21, 2020. Accessed March 13, 2021. Computable resource at: https://fevir.net/resources/Evidence/55.	A Fast Healthcare Interoperability Resources (FHIR) Resource of type Evidence that provides statistical and qualitative findings from meta‐analysis of three randomized trials evaluating the effect of Remdesivir on 14‐day mortality in patients with COVID‐19.

### 5.2 Category 1: Type metadata

Metadata describing CBKs by type are fundamental. Type metadata allow grouping and classifying of CBKs according to their most salient distinguishing characteristics. There is no established CBK typology of which we are aware and no single way to type CBKs. Type metadata elements tend to describe CBKs in the most general terms, for example, types that distinguish the information conveyed by CBKs (eg, value set, order set, computable phenotype, or computable guideline), types that distinguish the models conveyed by CBKs from one another (eg, predictive, risk, cost, cost‐benefit, risk, or causal models), or types that distinguish the form of expression (eg, document, executable code, message thread). As one real‐world example of CBK typing, in the AHRQ CDS Connect repository, CBK types include event‐condition‐action rule, risk assessment, order set, and multimodal CBKs, among others.

In our examples of CBK type metadata, we exclusively use the is_a predicate. Below are two examples of CBK type metadata in the following format [CBK] ***is_a*** {type}. Type metadata similar to the examples below are likely to be important for robust CBK search capabilities. Finding CBKs by type or excluding other CBKs by type are both supported by type metadata.

EXAMPLES OF TYPE METADATA

[CBK Example A] ***is_a*** {event‐condition‐action rule}.

[CBK Example B] ***is_a*** {predictive model}

### 5.3 Category 2: Domain metadata

Domain metadata indicate the subject of CBKs or what CBKs are about. CBK domain metadata support topical description of CBKs at many levels of abstraction. Hence, domain metadata can be general or highly specific. Domain metadata can be used to group and classify CBKs into one or more relevant biomedical domains or topic areas (eg, cancer). There is no single way to describe the domains of CBKs. There are many terminologies that could be used for this. Two potentially useful terminologies are Medical Subject Headings (ie, MeSH terms) and the Gencode Encyclopedia of Genes and Gene Variants.

To generate our domain metadata examples, we exclusively use the ***is_about*** predicate. Below are three examples of CBK domain metadata in the following format [CBK] ***is_about*** {domain (term ID)}. Domain metadata similar to the examples below are also likely to be important for CBK search. These metadata facilitate including or excluding CBKs according to their relevance to a domain of interest.

EXAMPLES OF DOMAIN METADATA

[CBK Example A] ***is_about*** {heart diseases (MeSH D006331)}.

[CBK Example A] ***is_about*** {lipid modifying agents (ATC1‐4 C10)}

[CBK Example B] ***is_about*** {using continuous vectors to represent protein sequences}

### 5.4 Category 3: Purpose metadata

Purpose metadata describe what the CBK is intended to be used for. In other words, purpose metadata provide answers to the question, “For what reasons was this CBK created?” Purpose metadata could be generated by the creators of a CBK at the time of its creation but do not have to be. Other CBK stakeholders and users can become “purpose‐givers” by declaring and documenting purposes throughout the CBK lifecycle.

Purpose metadata and the CBK uses they describe can be broad or narrow in scope. Broad purposes for the CBK might include using the CBK for “pilot testing” or “clinical decision support.” An example of a much narrower CBK purpose could be “provide a step‐by‐step workflow for glaucoma treatment management in the context of primary care.” Purpose metadata can also be used to place limitations on CBK use by declaring what the CBK is not intended for.

For purpose metadata, we suggest several predicates that convey purposes or intents such as ***has_purpose***, ***is_intended_to***, and ***is_not_intended_to***. Below are several examples of CBK purpose metadata. Purpose metadata similar to the examples below are also likely to be important for CBK search. These metadata can help searchers find CBKs with purposes of interest to them.

EXAMPLES OF PURPOSE METADATA

[CBK Example A] ***has_purpose*** {clinical decision support}.

[CBK Example A] ***is_intended_to*** {provide patient‐centered, evidence‐based preventive health information to patients between 40‐75 years old who have one or more cardiovascular disease (CVD) risk factor and a 10‐year CVD event risk score of 10% or greater}

[CBK Example A] ***is_not_intended_to*** {provide health information about children}

[CBK Example B] ***is_intended_to*** {predict relevant sequence features for single protein sequences}

### 5.5 Category 4: Identification metadata

Findability requires both location metadata (covered by the Location category) to determine “where” to find the CBK and identification metadata to “recognize” the CBK. Identification metadata may support findability by using identifiers in the search parameters (“Find me the item with this exact title.”) or in the search results (“Identify all the items found that match my query.”)

Identification metadata may include a variety of names, titles, and labels and may be derived from or include a combination of identification elements. To support reuse within and across systems, identifiers may be unique (UID), universally unique (UUID), and persistent and unique (PUID). To support interoperability, identifier metadata may include metadata elements to represent the identification system in addition to the identifier itself.

Findability hinges on having reliable PUIDs and other stable identification metadata. Identification metadata are critical to distinguish CBKs and their versions from each other. For this reason, versioning metadata is included here as a subcategory of identification metadata.

To generate examples of identification metadata, we use predicates that specify identifiers and versions. Below are several examples of CBK identifier metadata.

EXAMPLES OF IDENTIFICATION METADATA

[CBK Example A] ***has_name*** {Statin Use for the Primary Prevention of CVD in Adults: Patient‐Facing CDS Intervention}.

[CBK Example A] ***has_version*** {0.1}

[CBK Example B] ***has_name*** {embedding2structure}

[CBK Example C] ***has_identifier*** {10.15490/FAIRDOMHUB.1.MODEL.640.1} {of identifier type DOI}

[CBK Example C] ***has_version*** {1}

### 5.6 Category 5: Location metadata

For accessibility, the most basic and necessary metadata must convey places where CBKs can be found and retrieved by users. Access to CBKs can be, but need not always be, via network access over the World Wide Web (WWW). While WWW network access to CBKs is very convenient, some CBKs may be so sensitive or complex that online access is not feasible. Therefore, the scope of location metadata needs to be broad enough to include online and physical locations.

To generate examples of location metadata, we use two similar predicates, ***has_location*** and ***is_located_at***. Below are three examples of location metadata. Note that a single CBK may have more than one virtual or physical location. Copies of CBKs may be considered separate distinct objects or duplicate instances of the same object. Some CBK preservation strategies are predicated on having multiple copies of CBKs in multiple locations.

EXAMPLES OF LOCATION METADATA

[CBK Example A] ***has_location*** {https://cds.ahrq.gov/cdsconnect/artifact/statin-use-primary-prevention-cvd-adults-patient-facing-cds-intervention}.

[CBK Example B] ***is_located at*** {kipoi.org}

[CBK Example B] ***is_located at*** {Technical University of Munich}

[CBK Example C] ***has_location*** {http://doi.org/10.15490/FAIRDOMHUB.1.MODEL.640.1}

### 5.7 Category 6: CBK‐to‐CBK relationship metadata

Knowledge is relational by nature^64^, ^65^, ^66^ and this is demonstrated by compound or multipart examples of shareable CBKs. For example, some “CDS artifacts” in the AHRQ CDS Connect repository^35^ combine event‐condition‐action rules (a type of CBK) with value sets (another type of CBK) to form individual instances of working CDS interventions with multiple CBK parts. We anticipate complex combinations of CBKs being used to form compound CBKs, vast collections of CBKs curated according to some curation logic, and multiplex semantic networks describing complex webs of relationships between CBKs. CBK‐to‐CBK relationship metadata is fundamental to compound CBKs, CBK collections, and semantic CBK networks.

There are potentially thousands of useful relationships between CBKs that may ultimately need to be described using metadata. Therefore, unlike the previous CBK metadata categories, the space of potential predicates for CBK‐to‐CBK relationship metadata is vast and mostly uncharted.

To generate a few early examples of CBK‐to‐CBK relationship metadata, we focus on relationships about modification or derivation, predecessors and successors, and combination use. The predicates we used for this are ***is_modification_of***, ***is_predecessor_of***, ***is_successor_of***, and ***is_used_with***. We view these examples as starting points toward further specifying a wide array of CBK‐to‐CBK relationship metadata with many different predicates. While CBK‐to‐CBK relationship metadata may support many aspects of FAIRness and trustability, in this initial formulation, we see these metadata as being particularly important for enhancing CBK interoperability. This is because interoperability is about how well two or more things work together.

EXAMPLES OF CBK‐TO‐CBK RELATIONSHIP METADATA

[CDC Anthrax Post‐Exposure Version 0.1] ***is_predecessor_of*** {CBK Example D}.

[CBK Example D] ***is_successor_of*** {CDC Anthrax Post‐Exposure Version 0.1}

[CBK Example B] ***is_used_with*** {http://kipoi.org/models/SeqVec/embedding/}

### 5.8 Category 7: Technical metadata

Technical metadata is another category that has a wide scope. This category spans the technical characteristics of individual CBKs, which are many and complex. Since CBKs are meant to be processed or executed by digital computers, technical metadata are needed to convey information that supports CBK processing or execution.

To generate some useful examples of technical metadata for CBKs, we focus on file types and sizes, technical dependencies, and inputs. These technical features of CBKs are described in example metadata using appropriate predicates.

EXAMPLES OF TECHNICAL METADATA

[CBK Example B] ***has_file_type*** {.py}.

[CBK Example B] ***has_file_size*** {4.47 kb}

[CBK Example B] ***has_dependency*** {Python 3.6}

[CBK Example B] ***has_input*** {numpy array}

### 5.9 Category 8: Authorization and rights management metadata

In the list of metadata categories spanning metadata to make CBKs FAIR+T, we have combined authorization metadata together with rights management metadata. Our view is that authorization is an important and special class of rights, including the rights to view (or access), comment on, or modify CBKs. Other rights related to CBKs may be specified as copyrights or through various software and other licenses. We also include metadata that assign specific responsibilities to individuals or organizations in this category and leave room for metadata about disclaimers too.

To generate realistic examples of authorization and rights management metadata, we use several predicates such as ***is_available_to***, ***has_license***, ***copyright_is_held_by***, and ***has_disclaimer***. Below are three examples of CBK authorization and rights management metadata. These metadata are key for CBK reusability because they provide information about the legal status of CBKs and the rights and responsibilities of CBK creators and users.

EXAMPLES OF AUTHORIZATION AND RIGHTS MANAGEMENT METADATA

[CBK Example A] ***copyright_is_held_by*** {United States Preventive Services Task Force (USPSTF)}.

[CBK Example A] ***has_license*** {AHRQ Government Unlimited Usage Rights}

[CBK Example B] ***has_license*** {MIT License}

### 5.10 Category 9: Preservation metadata

Preservation metadata represent the information needed for the conservation of CBKs over decades. Preservation metadata support long‐term archiving by indicating aspects like the planned duration of archiving and by specifying various methods of digital preservation. These metadata have a special role to play in support of root cause analyses of incidents involving CBKs, sometimes long after CBKs have been taken out of use. Preservation metadata also support the safekeeping of CBKs for future research.

Rather than start from scratch, for our examples of preservation metadata, we draw on two predicates from the preservation metadata: implementation strategies (PREMIS) ontology.^67^ These predicates are ***has_preservation_level*** and ***should_be_kept_until***. According to PREMIS, achieving a preservation level of “medium” means two copies of a CBK are stored on different media types with a minimum of 150 km distance between the two stored copies, with separate checksums checked annually. Since long‐term access to CBKs directly supports their reuse, we associate preservation metadata most strongly with reusability.

EXAMPLES OF PRESERVATION METADATA

[CBK Example A] ***has_preservation_level*** {Medium}.

[CBK Example B] ***should_be_kept_until*** {January 1, 2040}

### 5.11 Category 10: Integrity metadata

As noted above, CBKs may be widely distributed over computer networks, including the WWW. In network environments, integrity metadata are used by senders and receivers to verify CBK authenticity and completeness. Cryptographic hash functions provide a mechanism that allows fetched CBKs to be checked for tampering that may have occurred during CBK network transit from sender to receiver.

For the most part, integrity metadata are processed by machines and not by people. An existing specification for integrity metadata is available from the W3C.^58^ Integrity metadata elements prevent unwarranted manipulation of CBKs, and thus they directly support trust in CBKs. We provide two examples of integrity metadata below.

EXAMPLES OF INTEGRITY METADATA

[CBK Example B] ***has_hash*** {de6ea2f798397aa7de1830da6cf88f5245faef1e0d09b10cf8e7c72929b17343}.

[CBK Example B] ***uses_has_function_type*** {SHA 256}

### 5.12 Category 11: Provenance metadata

Provenance metadata record key events in CBK lifecycles, including changes in ownership, custody, or composition of CBKs. Provenance metadata closely relate to versioning metadata, which we covered in the identification metadata category described above.

Provenance metadata may be fine‐ or coarse‐grained depending on the level of detail needed about the lifecycles of CBKs. We recognize the PROV‐O ontology and the support it provides for specifying complex provenance metadata.^59^

To generate some basic examples of provenance metadata for CBKs, we used the following predicates, ***is_owned_by*** {agent}, ***has_status*** {status}, and ***status_changed_on*** {date}. These provenance metadata convey a change that took place in the lifecycle of CBKs. Provenance metadata uphold trust by providing a mechanism to track and trace CBKs from their origin, through their period of use in practice, and up to their ultimate deprecation, withdrawal, and deletion.

Provenance metadata may also include responsibilities for the content of CBK and could describe *contributorship*, including who contributed, what they contributed, and when they contributed to the CBK content. For simplicity in our examples, we used predicates named for common contributor roles, ***is_authored_by*** {author}, ***is_reviewed_by*** {reviewer}, and ***is_endorsed_by*** {endorser}.

EXAMPLES OF PROVENANCE METADATA

[CBK Example A] ***is_owned_by*** {AHRQ}.

[CBK Example A] ***has_status*** {Active}

[CBK Example A] ***status_changed_on*** {June 1, 2019}

### 5.13 Category 12: Evidential basis metadata

Since CBKs convey knowledge, they are warranted by underlying evidence of some type, such as empirical evidence or expert opinion. We generally refer to any and all of this underlying evidence as the ***evidential basis*** of CBKs. Further, we recognize that prior work has gone into grading the evidence supporting knowledge claims for clinical practice guidelines.^68^, ^69^ With existing evidence grading approaches in mind, we also incorporate metadata about evidence grades into this evidential metadata category.

Furthermore, following the work of Lehmann and Downs that specified desiderata for shareable CBKs,^2^ we recognize the complexity of specifying aspects of the evidential basis of CBKs using metadata. We foresee the need for a substantial body of future work on evidential basis metadata for CBKs.

Here we make a small start by specifying several initial predicates of interest. Two examples of metadata constructed using those predicates are given below.

EXAMPLES OF EVIDENTIAL BASIS METADATA

[CBK Example A] ***is_based_on_data_collected_by*** {United States Preventive Services Task Force (USPSTF)}.

[CBK Example A] ***has_certainty_of_evidence*** {USPSTF Evidence Grade A}

### 5.14 Category 13: Evidence from use metadata

In direct contrast to evidential basis metadata, when put into use, the outcomes from using CBK is new and different evidence about them. This ***evidence from use*** relates the performance and real‐world impacts of CBK, and it can be conveyed by more metadata. A simple example of evidence from use metadata is metadata that describe who, what, when, where, and why CBKs are used. More sophisticated examples of evidence from use may arise from various evaluations for CBKs. This metadata category anticipates a world where CBKs are widely used and studied.

EXAMPLES OF EVIDENCE FROM USE METADATA

[CBK related to CBK Example A] ***use_is_evaluated_in*** {Conwell L, Barterian L, Rose A, Peterson G, Kranker K, Blue L, Magid D, Williams M, Steiner A, Sarwar R, Tyler J. Evaluation of the Million Hearts Cardiovascular Disease Risk Reduction Model: First Annual Report.}.

### 5.15 Application of metadata categories to real‐world CBKs

**FIGURE 1:**
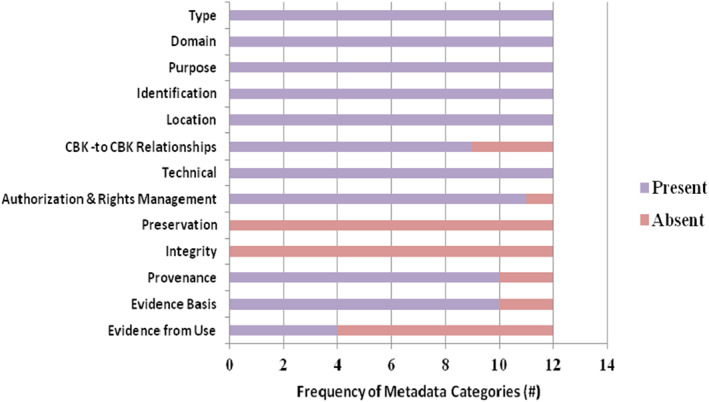
Available metadata by category for 12 existing CBKs found online

To check the current availability of metadata from the 13 metadata categories, we identified 12 CBKs available online and examined the existing metadata for each CBK in light of the categories. Summary information about the metadata we found by category is provided in Figure 1. In addition, a Supplement with this paper provides more details about these 12 CBKs and their metadata.

### 5.16 Research agenda for CBK metadata

Another result is the research agenda for future CBK metadata research (Table 3). This agenda emerged from our discussions of categories of metadata for making CBKs FAIR+T. Overall, we recognize that a significant body of additional research work needs to be completed to answer open questions about the metadata elements in each category of the CBK metadata categories in Table 1.

**TABLE 3:** Research agenda for further CBK metadata exploration and analysis

Research agenda item	Brief description of research agenda item	Related metadata category
CBK typologies	A variety of different approaches have been taken to define the types and subtypes of CBKs. More work is needed to synthesize these efforts into coherent CBK typologies to support standards for CBK types.	Type
Schema for purpose metadata	There is an apparent need to formalize CBK purpose metadata. As complex artificial artifacts, all CBKs emerge from some human design process. It may be possible to create schema to convey the motivations and intents of CBK designers and of CBK users and others coherently and usefully.	Purpose
Schema for CBK‐to‐CBK relationships metadata	The many ways in which CBKs relate to one another are not clear. Work is needed to examine potential relationships between types of CBKs and actual relationships between existing CBKs.	CBK‐to‐CBK relationships
CBK lifecycles	The lifecycles of CBKs need to be better understood. Since CBK lifecycles may vary by CBK type, interactions between Provenance Metadata and Type Metadata need to be explored.	Provenance, Type, Preservation
CBK use outcomes	It is not clear which outcomes from using CBKs are of most interest to users. Studies of CBK user needs for evidence arising from use of CBKs are needed to better understand outcomes of interest.	Evidence from Use
Relationships between CBK metadata and the FAIR and trustability principles	Studies to test the hypotheses surfaced here that metadata from 13 categories can uphold the findability, accessibility, interoperability, reusability, and trustability of CBKs are needed.	All

## 6 DISCUSSION

We envision a future in which CBKs are widely shared to support biomedical research, education, and improvement of individual and population health. A year of effort has resulted in a list of 13 metadata categories relevant for making CBKs FAIR+T. Having reviewed the metadata for a variety of actual CBKs, it seems likely that many CBK stakeholders will benefit from higher quality CBK metadata.

The list of categories should not be confused with a settled metadata framework, let alone a specification. Instead, we view this list of CBK metadata categories as the first step in a longer CBK metadata specification process. Next steps include gathering feedback toward achieving broad consensus for a draft CBK metadata framework and specification, including common elements and value sets for metadata in each category. We hope that by providing a list of potentially relevant metadata categories for making CBKs FAIR+T along with a research agenda, we have done enough to prompt further steps toward a common CBK metadata framework and future specification.

Metadata involve a variety of standards and models for their structure, syntax, content, and communication.^41^ We make use of certain existing metadata standards and models to offer examples (eg, Dublin Core, RDF). We do not put forward any new standard or model. Instead, we offer guidance about the scope of CBK metadata for future standards and model development. Likewise, while we recognize the importance of the metadata generation process, we do not address metadata generation for CBK. Instead, we limit our investigation to examining previously generated metadata about CBKs.

Our metadata categories list focuses primarily on the metadata needs and contributions of CBK producers and consumers (or users). When the value of specific metadata elements is demonstrated, we expect CBK producers will provide a minimum set of metadata to support CBK consumers. Some of this metadata, such as persistent unique identifiers and access locations, could be generated automatically.

The large scope of our metadata categories is a major concern. The costs of generating and managing sufficient CBK metadata to make CBKs FAIR+T could be high, potentially limiting widespread CBK mobilization, sharing, and use. The barriers to creating such metadata are high.^43^ Consequently, CBK producers and consumers need ways to minimize and recoup the costs of providing sufficient metadata. While producers need to supply most of the metadata to make CBKs FAIR, consumers must supply some metadata from their experience of CBK use to uphold trust.^5^ The value of every metadata element in each category needs to be determined to justify costs. For the sample of 12 CBKs that we inspected, we did not find any integrity or preservation metadata (see Figure 1), and we found little technical metadata giving instructions for CBK use. These metadata may be costlier to produce than others.

Two categories of metadata in the list are tentative—the “Purpose” category and the “CBK‐to‐CBK Relationships” category. We believe both these categories need to be further refined.

Two closely related efforts include FAIR principles for software development. In 2016, the Software Citation Working Group of the FORCE11 organization published its principles for software citation.^70^ Of their six principles, five relate to metadata content. These five principles uphold software metadata for attribution, identifiers, persistence and preservation, accessibility, and version specificity. The metadata in our 13 categories includes these principles. The authors of these five principles on software citation also discuss software types and distinguish between software that is accessible as source code and software that is only accessible as a service. Adding to these ideas, in mid‐2020, a group allied with the Research Data Alliance published the paper, toward FAIR Principles for Research Software.^71^ As we do in this work, these authors also ground their efforts to make research software FAIR by evoking the notion of FAIR Digital Objects. They stipulate that research software is not data and argue that making software FAIR will require a software‐specific approach like the approach pioneered in this manuscript.

Finally, we see linkages between this work on CBK metadata and some other major initiatives. For example, the Agency for Healthcare Research and Quality evidence‐based Care Transformation Support (ACTS) initiative and the Center for Reproducible Biomedical Modeling both represent efforts at the federal level in the U.S. to advance CBK sharing in part by specifying and using CBK metadata. Also, the Fast Healthcare Interoperability Resources (FHIR) standard established by Health Level 7 International (HL7) for CBKs in the health domain is being extended to the research domain.^72^ These developments connecting CBKs across vast domains offer technical and organizational opportunities to develop common metadata frameworks across wide‐reaching CBK spaces.

## 7 LIMITATIONS

The main limitations of this work are its consensus‐based approach and the small number of real‐world CBKs examined. Consensus among a small group is not predictive of consensus among a much larger group of stakeholders.

We had only enough input to work on metadata categories and did not specify the metadata elements in each category. We do not believe that one set of metadata elements will suffice to describe all CBKs. Our explorations show that many different types of CBKs already exist, and that their metadata vary by type. In addition, although complex hierarchical sets of metadata assertions are sometimes required (such as system specification for identifiers or codes), we limited our examples to simple metadata assertions (presented wholly as independent triples). This will not suffice for a future specification.

There still exists some conceptual overlap among our categories. For example, the “Type” and “Technical” metadata categories overlap. If CBK typing is done based on technical differences, then these two categories blur. However, it is well established that all categorization schemes are imperfect and incomplete.^73^

As a strategy to mobilize CBK, we look forward to further developing and refining our CBK metadata categories list and to learning more about CBK metadata from the real‐world experiences of researchers, educators, clinicians, and other consumers who use CBKs in their work.

## 8 CONCLUSION

Computable biomedical knowledge artifacts (CBKs) vary widely in their complexity, goals, and anticipated audience. Each CBK offers knowledge of potential value for clinical care, public health, education, or for advancing biomedical science. Sharing of complex CBKs is key to support *systems biology*, *precision health*, *population health*, and *learning health system* initiatives.

To mobilize CBKs effectively, the value from sharing CBKs has to be greater than the costs of sharing them. For producers of CBKs, easier ways to disseminate CBKs to those able to benefit is of prime importance. For consumers of CBKs, the ability to readily discover, deploy, and use CBKs to meet their clinical, educational, or scientific needs is most important.

Ultimately, a common metadata framework for CBKs can advance efforts to mobilize CBKs. As an initial step, we contribute a list of 13 metadata categories for making CBKs findable, accessible, interoperable, reusable, and trustable (FAIR+T).

## CONFLICT OF INTEREST

Brian S. Alper owns Computable Publishing LLC. Mark Tuttle is on the Board of Directors for Apelon and has an equity position. Gunes Koru owns Maryland Health Information Technology LLC and Maryland Data Science and Engineering LLC. No conflicts of interest were reported by Allen Flynn, Bruce E. Bray, Marisa L. Conte, Christina Eldredge, Sigfried Gold, Robert A. Greenes, Peter Haug, Kim Jacoby, James McClay, Marc Sainvil, Davide Sottara, Shyam Visweswaran, and Robin Ann Yurk.

## Supporting information


**Appendix S1** Supporting information
