# Supplementary material for: Categorizing metadata to help mobilize computable biomedical knowledge
Source: Learn Health Syst. 2021 May 9;6(1):e10271. doi: 10.1002/lrh2.10271 (PMC8753304; doi:10.1002/lrh2.10271)
Supplement: Supplementary file 1 — Appendix S1 Supporting information [file LRH2-6-e10271-s001.docx]

**SUPPLEMENT**

**Categorizing Metadata to Help Mobilize Computable Biomedical Knowledge**

**Application of Newly Developed Metadata Categories List to**

**12 Actual CBK Examples Found Online Labeled A through L**

**CBK Example A – Clinical Decision Support Rule**

| METADATA  CATEGORY | EXISTING METADATA |
| --- | --- |
| 1. Type | + is a Event-Condition-Action (ECA) rule  + is a producer of patient-facing output |
| 2. Domain | + is about clinical care  + is about heart diseases (MeSH D006331)  + is about lipid modifying agents (ATC1-4 C10)  + is about cardiovascular disease risk |
| 3. Purpose | + has purpose clinical decision support  + has purpose primary prevention  + has purpose to prompt patients to discuss possible statin therapy with their physicians  + is intended to provide patient-centered, evidence-based preventive health information to patients  between 40-75 years old who have one or more cardiovascular disease (CVD) risk factor and a 10  year CVD event risk score of 10% or greater  + is not intended to provide health information about children  + is for adults of chronological age in the range of 40 to 75 years  + intended for adults 40 years and older without a history of CVD who do not have current signs and  symptoms of CVD (i.e., symptomatic coronary artery disease or ischemic stroke). |
| 4. Identification | + has name Statin Use for the Primary Prevention of CVD in Adults: Patient-Facing CDS Intervention  + has title Statin Use for the Primary Prevention of CVD in Adults: Patient-Facing CDS Intervention  + has version 0.1 |
| 5. Location | + has location <https://cds.ahrq.gov/cdsconnect/artifact/statin-use-primary-prevention-cvd-adults-patient-facing-cds-intervention>  + has location AHRQ CDS Connect |
| 6. CBK-to-CBK   Relationships | + is companion to Statin Use for the Primary Prevention of CVD in Adults: Clinician-Facing CDS  Intervention  + uses CMS’s Million Hearts® Model Longitudinal ASCVD Risk Assessment Tool for Updated 10-Year ASCVD Risk |
| 7. Technical | + is encoded in Clinical Quality Language (CQL)  + is expressed using Clinical Quality Language (CQL)  + has implementation guide  + executed using CQL Services at <https://github.com/AHRQ-CDS/AHRQ-CDS-Connect-CQL-SERVICES> |
| 8. Authorization & Rights Management | + has license AHRQ Government Unlimited Usage Rights  + copyright is held by USPSTF  + copyright is administered by AHRQ |
| 9. Preservation | (Example in the manuscript text is fictitious. None found with the actual CBK.) |
| 10. Integrity | (Example in the manuscript text is fictitious. None found with the actual CBK.) |
| 11. Provenance | + is owned by AHRQ  + has steward AHRQ  + published by The MITRE Corporation  + has status Active  + created on June 1, 2019  + status changed on June 1, 2019  + last review date September 8, 2019 |
| TWO EVIDENCE CATEGORIES | |
| 12. Evidential   Basis | + is based on data collected by United States Preventive Services Task Force (USPSTF)  + has strength of evidence USPSTF Evidence Grade A  + is based on United States Preventive Services Task Force. 2016. Statin Use for the Primary  Prevention of CVD in Adults. Journal of the American Medical Association. November 16, 2016.  Volume 316, Number 19. Accessed on April 10, 2016 |
| 13. Evidence   from Use | NOTE: A related CBK (CMS’s Million Hearts® Model Longitudinal ASCVD Risk Assessment Tool for Updated 10-Year ASCVD Risk) has Evidence from Use: Conwell L, Barterian L, Rose A, Peterson G, Kranker K, Blue L, Magid D, Williams M, Steiner A, Sarwar R, Tyler J. Evaluation of the Million Hearts® Cardiovascular Disease Risk Reduction Model: First Annual Report. |

CITATION: Statin Use for the Primary Prevention of CVD in Adults: Patient-Facing CDS Intervention [Clinical Decision Support Artifact], version 0.1. Contributors: The MITRE Corporation, US Preventive Services Task Force [Contributors], Agency for Healthcare Research and Quality [Steward]. In: CDS Connect. Created June 1, 2019. Approved September 8, 2019. Accessed December 5, 2020. Available at: https://cds.ahrq.gov/cdsconnect/artifact/statin-use-primary-prevention-cvd-adults-patient-facing-cds-intervention.

COMMENTS: This example is mentioned in the manuscript. Credit for metadata table: Greenes, Flynn

**CBK Example B – Predictive Model**

| METADATA  CATEGORY | EXISTING METADATA |
| --- | --- |
| 1. Type | + is a predictive model  + is a 3-state, 8-state secondary structure and disorder prediction model based on SeqVec  + is a Dataset |
| 2. Domain | + is about using continuous vectors to represent protein sequences + is about bioinformatics  + is about proteins |
| 3. Purpose | + has intent predict relevant sequence features for single protein sequences |
| 4. Identification | + has name embedding2structure |
| 5. Location | + is located at kipoi.org  + is located at Technical University of Munich  + has location <http://kipoi.org/models/SeqVec/embedding2structure/> |
| 6. CBK-to-CBK   Relationships | + is used with <http://kipoi.org/models/SeqVec/embedding/> |
| 7. Technical | + has file type .py + has file size 4.47kb + has dependency Python 3.6  + has dependency scikit-learn==0.22.2.post1  + has dependency conda-forge::allennlp  + has input numpy array |
| 8. Authorization & Rights Management | + has license MIT license |
| 9. Preservation | (Example in the manuscript text is fictitious. None found with the actual CBK.) |
| 10. Integrity | (Example in the manuscript text is fictitious. None found with the actual CBK.) |
| 11. Provenance | + contributed by Michael Heinzinger  + author Michael Heinzinger |
| TWO EVIDENCE CATEGORIES | |
| 12. Evidential   Basis | +is described at <https://www.biorxiv.org/content/10.1101/614313v3> |
| 13. Evidence   from Use |  |

CITATION: SeqVec/embedding2structure [Model]. Contributor: Michael Heinzinger [Author]. In: Kipoi.org, doi 10.1101/614313. Accessed December 5, 2020. Available at: http://kipoi.org/models/SeqVec/embedding2structure/. Computable resource at: <https://github.com/kipoi/models/tree/master/SeqVec/embedding2structure>.

COMMENTS:

This example is mentioned in the manuscript.

Credit for metadata table: Flynn

**CBK Example C – Model of Ordinary Differential Equations**

| METADATA  CATEGORY | EXISTING METADATA |
| --- | --- |
| 1. Type | + is a model of ordinary differential equations  + has type ODE  + systems biology model |
| 2. Domain | + is about inflammation  + is inflammatory environments  + is about Predictable Irreversible Switching Between Acute and Chronic Inflammation  + is about a computational systems biology model of IgE-induced inflammation |
| 3. Purpose | + has purpose to model IgE-mediated mast-cell activation and a relatively small number of factors and  interactions so that the model’s results can also be understood more intuitively + has purpose to provide a way to deal with the complexity of IgE-induced inflammation regulation by  making predictive models and by simulation in silico. |
| 4. Identification | + has name Innate Inflammation; model 2018  + has version 1  + has model number FAIRDOM Hub 640  + has identifier <http://doi.org/10.15490/FAIRDOMHUB.1.MODEL.640.1>  + has SEEK ID <https://fairdomhub.org/models/640?version=1>  + has citation Westerhoff, H. (2019). Innate Inflammation; model 2018. FAIRDOMHub.  http://doi.org/10.15490/FAIRDOMHUB.1.MODEL.640.1 |
| 5. Location | + has location <https://fairdomhub.org/models/640>  + has location FAIRDOM Hub |
| 6. CBK-to-CBK   Relationships |  |
| 7. Technical | + has model format Copasi  + has file format code cps  + has file format XML  + has file size 103Kb  + has execution or visualization environment Copasi |
| 8. Authorization & Rights Management | + has license Creative Commons Attribution 4.0 |
| 9. Preservation |  |
| 10. Integrity |  |
| 11. Provenance | + has creators Hans Westerhoff, Ablikim Abudukelimu  + has submitter Hans Westerhoff  + created on November 5, 2019 at 15:18 GMT |
| TWO EVIDENCE CATEGORIES | |
| 12. Evidential   Basis | + is described in Abudukelimu, A., Barberis, M., Redegeld, F.A., Sahin, N., and Westerhoff, H.V.  (2018). Predictable Irreversible Switching Between Acute and Chronic Inflammation. Front Immunol  9, 1596. |
| 13. Evidence   from Use | + has views 385  + has downloads 45  + last used on December 6, 2020 at 13:03 GMT |

CITATION: Innate Inflammation; model 2018 [Model], version 1. Contributors: Hans Westerhoff [Contributor, Submitter], Ablikim Abudukelimu [Contributor]. In: FAIRDOM Hub, model 640. Created November 5, 2019. Accessed December 6, 2020. Available at: https://fairdomhub.org/models/640. Computable resource at: https://fairdomhub.org/models/640/download?version=1.

COMMENTS:

This example is mentioned in the manuscript.

Credit for metadata table: Flynn

**CBK Example D – Clinical Decision Support Logic**

| METADATA  CATEGORY | EXISTING METADATA |
| --- | --- |
| 1. Type | + is a multimodal artifact  + is a experimental artifact  + is a clinical decision support artifact |
| 2. Domain | + is about clinical care  + is about emergency medicine  + is about anthrax  + is adult anthrax treatment  + is about anthrax post-exposure prophylaxis (PEP) |
| 3. Purpose | + has purpose to provide clinicians with information for quickly assessing individuals exposed to  anthrax unrelated to occupational exposure, for example in bioterrorism events, and provides the  recommended post-exposure prophylaxis (PEP) regimen for asymptomatic patients.  + has purpose clinical decision support  + has intended population adults ≥18 years of age exposed to anthrax unrelated to occupational  exposure.  + intended for use by clinicians caring for individuals ≥18 years of age in an outpatient setting. |
| 4. Identification | + has name Anthrax Post-Exposure Prophylaxis  + has title Anthrax Post-Exposure Prophylaxis  + has version 0.3 |
| 5. Location | + has location <https://cds.ahrq.gov/cdsconnect/artifact/anthrax-post-exposure-prophylaxis>  + has location AHRQ CDS Connect |
| 6. CBK-to-CBK   Relationships | + has predecessor CDC Anthrax Post-Exposure Version 0.1  + is successor of CDC Anthrax Post-Exposure Version 0.1 |
| 7. Technical | + logic tested using 61 automated tests  + is encoded in Clinical Quality Language (CQL)  + is expressed using Clinical Quality Language (CQL)  + has implementation guide |
| 8. Authorization & Rights Management | + has license Apache  + copyright is held by The MITRE Corporation |
| 9. Preservation |  |
| 10. Integrity |  |
| 11. Provenance | + has status Draft  + has steward Centers for Disease Control and Prevention (CDC)  + published by Centers for Disease Control and Prevention (CDC)  + created on October 25, 2018  + status changed on October 25, 2018  + last review date August 6, 2020 |
| TWO EVIDENCE CATEGORIES | |
| 12. Evidential   Basis | + is based on Centers for Disease Control and Prevention anthrax guidelines and Advisory Committee  on Immunization Practices anthrax vaccination guidelines |
| 13. Evidence   from Use | + not tested in a live clinical setting |

CITATION: Anthrax Post-Exposure Prophylaxis [Clinical Decision Support Artifact], version 0.2. Contributors: The MITRE Corporation [Contributor], Centers for Disease Control and Prevention [Steward]. In: CDS Connect. Created October 25, 2018. Approved August 6, 2020. Accessed December 5, 2020. Available at: https://cds.ahrq.gov/cdsconnect/artifact/anthrax-post-exposure-prophylaxis.

COMMENTS:

This example is mentioned in the manuscript.

Credit for metadata table: Flynn

**CBK Example E – Risk Score based on Equation**

| METADATA  CATEGORY | EXISTING METADATA |
| --- | --- |
| 1. Type | + is an implementation of a mathematical equation  + is a risk calculator  + is a predictive model |
| 2. Domain | + is about clinical care  + is about risk assessment  + is about cardiovascular disease risk  + is about patient education |
| 3. Purpose | + is intended for people between the ages of 40 and 79. It helps predict your risk over 10 years of heart  attack, stroke, or death from cardiovascular disease.  + is intended to educate patients on their cardiovascular disease risk |
| 4. Identification | + has title Cardiovascular risk assessment in adults (10-year, ACC/AHA 2013) (Patient education)  + has version 3.0  + has topic number 119179 |
| 5. Location | + has location <https://www.uptodate.com/contents/calculator-cardiovascular-risk-assessment-in-adults-10-year-acc-aha-2013-patient-education>  + has location UpToDate ® |
| 6. CBK-to-CBK   Relationships |  |
| 7. Technical | + is implemented as web form  + has inputs of race, sex, age, total cholesterol level, HDL level, systolic blood pressure, use of blood  pressure medication status, smoking status, diabetes status  + has output of estimate of risk of heart attack, stroke or death from cardiovascular event over 10 years |
| 8. Authorization & Rights Management | + copyright is held by Foundation Internet Services, LLC |
| 9. Preservation |  |
| 10. Integrity |  |
| 11. Provenance |  |
| TWO EVIDENCE CATEGORIES | |
| 12. Evidential   Basis | + is based on Goff DC Jr, Lloyd-Jones DM, Bennett G, et al. 2013 ACC/AHA Guideline on the  Assessment of Cardiovascular Risk: A Report of the American College of Cardiology/American Heart  Association Task Force on Practice Guidelines. Circulation 2014; 129:S49.  + is based on data from White American and African American people |
| 13. Evidence   from Use |  |

CITATION: Calculator: Cardiovascular risk assessment in adults (10-year, ACC/AHA 2013) (Patient education) [Interactive Form], version 3.0. In: EBMcalc in UpToDate, Topic 119179. Accessed December 5, 2020. Available at: https://www.uptodate.com/contents/calculator-cardiovascular-risk-assessment-in-adults-10-year-acc-aha-2013-patient-education.

COMMENTS:

Credit for metadata table: Flynn

**CBK Example F – Risk Score based on Point Score**

| METADATA  CATEGORY | EXISTING METADATA |
| --- | --- |
| 1. Type | + is a risk calculator  + is a risk assessment tool  + implements a point-scoring system |
| 2. Domain | + is about stroke  + is about thromboembolic stroke  + is about risk assessment  + is about 1-year risk of a thromboembolic stroke in patients with Atrial Fibrillation (MESH: D001281) |
| 3. Purpose | + is intended to assess long-term stroke risk in a non-anticoagulated patient with non-valvular Atrial  Fibrillation  + is intended to implement the CHA2DS2-VASc score |
| 4. Identification | + has title CHA₂DS₂-VASc Score |
| 5. Location | + has location <https://www.mdcalc.com/cha2ds2-vasc-score-atrial-fibrillation-stroke-risk>  + has URL <https://www.mdcalc.com/cha2ds2-vasc-score-atrial-fibrillation-stroke-risk> |
| 6. CBK-to-CBK   Relationships | + is successor to CHADS₂ Score |
| 7. Technical | + is implemented as web form or mobile application  + reports risk per year  + has inputs of age, sex, CHF history, hypertension history, stroke history, vascular disease history,  diabetes history  + has output of estimate of risk of heart attack, stroke or death from cardiovascular event over 10 years |
| 8. Authorization & Rights Management | + copyright is held by MDCalc  + has terms of use <https://www.mdcalc.com/terms> |
| 9. Preservation |  |
| 10. Integrity |  |
| 11. Provenance | + risk score created by Gregory Lip, MD  + CBK author (listed as content contributor) Calvin Hwang, MD  + is owned by MDCalc |
| TWO EVIDENCE CATEGORIES | |
| 12. Evidential   Basis | + is based on 1,084 patients with non-valvular AF, not on anticoagulation, over age 18 with EKG or  Holter diagnosed AF in the ambulatory and hospital settings from 182 hospitals in 35 countries from  2003 to 2004 and had known thromboembolic status at 1 year from the Euro Heart Survey database.  + is based on <https://www.ncbi.nlm.nih.gov/pubmed/22922413>  + validated in <https://www.ncbi.nlm.nih.gov/pubmed/23408865>  + validated in <https://www.ncbi.nlm.nih.gov/pubmed/22246443>  + validated in <https://www.ncbi.nlm.nih.gov/pubmed/24759791> |
| 13. Evidence   from Use | + has evidence summary statement Numerous validation studies have shown that CHA2DS2-VASc is  as good as - or possibly better - than CHADS2 at predicting high risk patients, but CHA2DS2-VASc  is certainly best at predicting the “low risk” patients. |

CITATION: CHA₂DS₂-VASc Score for Atrial Fibrillation Stroke Risk [Interactive Form]. Contributors: Calvin Hwang [Content Contributor], Gregory Lip [Creator of risk score]. In: MDCalc platform. Created September 17, 2009. Accessed March 14, 2021. Available at: https://www.mdcalc.com/cha2ds2-vasc-score-atrial-fibrillation-stroke-risk.

COMMENTS:

Credit for metadata table: Jacoby, Flynn

**CBK Example G – Value Set**

| METADATA  CATEGORY | EXISTING METADATA |
| --- | --- |
| 1. Type | + is a value set  + is a grouping value set |
| 2. Domain | + is about coding condition or diagnosis  + is about diabetes |
| 3. Purpose | + is intended to support electronic clinical quality measurement  + has purpose to identify patients who have a diagnosis of diabetes  + not intended for patients who have gestational diabetes or steroid-induced diabetes |
| 4. Identification | + has title Diabetes  + has identifier of type OID  + has identifier 2.16.840.1.113883.3.464.1003.103.12.1001 |
| 5. Location | + has location <https://vsac.nlm.nih.gov/valueset/2.16.840.1.113883.3.464.1003.103.12.1001/expansion/Latest>  + has location Value Set Authority Center (VSAC) |
| 6. CBK-to-CBK   Relationships | + includes the union of codes from   Other Diabetes ICD10CM 2.16.840.1.113883.3.464.1003.103.11.1032,  Other Diabetes ICD9CM 2.16.840.1.113883.3.464.1003.103.11.1033,   Type 1 Diabetes ICD9CM 2.16.840.1.113883.3.464.1003.103.11.1025,   Type 1 Diabetes ICD10CM 2.16.840.1.113883.3.464.1003.103.11.1026,  Type 1 Diabetes SNOMEDCT 2.16.840.1.113883.3.464.1003.103.11.1027,  Type II Diabetes SNOMEDCT 2.16.840.1.113883.3.464.1003.103.11.1030,  Type II Diabetes ICD10CM 2.16.840.1.113883.3.464.1003.103.11.1031,  Type II Diabetes ICD9CM 2.16.840.1.113883.3.464.1003.103.11.1034 |
| 7. Technical | + includes 421 coded concepts  + has coded concepts from SNOMED-CT  + has coded concepts from ICD9CM  + has coded concepts from ICD10CM  + has file format .xls |
| 8. Authorization & Rights Management | + authorization requires UMLS account  + authorization managed by UMLS Terminology Services  + has license UMLS license |
| 9. Preservation | + VSAC preserves all past versions of a value set |
| 10. Integrity |  |
| 11. Provenance | + has steward NCQA |
| TWO EVIDENCE CATEGORIES | |
| 12. Evidential   Basis |  |
| 13. Evidence   from Use | + validated in Weiskopf NG, Cohen AM, Hannan J, Jarmon T, Dorr DA. Towards augmenting  structured EHR data: a comparison of manual chart review and patient self-report. AMIA Annu Symp  Proc. 2020 Mar 4;2019:903-912. PMID: 32308887; PMCID: PMC7153078. |

CITATION: Diabetes [Terminology], version 20190315. Contributors: National Committee for Quality Assurance [Steward]. In: Value Set Authority Center, OID 2.16.840.1.113883.3.464.1003.103.12.1001. Accessed October 27, 2020. Available at: https://vsac.nlm.nih.gov/valueset/2.16.840.1.113883.3.464.1003.103.12.1001/expansion/Latest [Login required]. Computable resource with: API or Excel export.

COMMENTS:

Credit for metadata table: Gold, Eldredge, Flynn

**CBK Example H – Computable Phenotype**

| METADATA  CATEGORY | EXISTING METADATA |
| --- | --- |
| 1. Type | + is a computable phenotype  + is preimplementation pseudocode  + e-algorithm for search |
| 2. Domain | + is about Familial Hypercholesterolemia |
| 3. Purpose | + is intended to identify patients with familial hypercholesterolemia programmatically using EHR data |
| 4. Identification | + has title Electronic Health Record-based Phenotyping Algorithm for Familial Hypercholesterolemia  + has version 2.0 |
| 5. Location | + has location <https://phekb.org/sites/phenotype/files/FH_eAlgorithm_Pseudocode_FullText_2016_1_3.pdf>  + has URL <https://phekb.org/sites/phenotype/files/FH_eAlgorithm_Pseudocode_FullText_2016_1_3.pdf>  + has location phekb.org |
| 6. CBK-to-CBK   Relationships |  |
| 7. Technical | + has format PDF  + has coded concepts from CPT-4  + has coded concepts from ICD9CM  + has coded concepts from LOINC  + has coded concepts from RxNorm |
| 8. Authorization & Rights Management |  |
| 9. Preservation |  |
| 10. Integrity |  |
| 11. Provenance | + owned by Mayo Clinic  + created during June, 2016  + authored by Iftikhar Kullo, MD, Adelaide Arruda-Olson, MD, PhD, Carin Smith,  Hongfang Liu, PhD, Majid Rastegar, Maya Safarova, MD, PhD, Parvathi Balachandran, MBBS, Saeed Mehrabi, Sunghwan Sohn, PhD, Xiao Fan, PhD, Yijing Cheng  + has repository record creator Maya Safarova |
| TWO EVIDENCE CATEGORIES | |
| 12. Evidential   Basis | + validated in Safarova MS, Liu H, Kullo IJ. Rapid identification of familial hypercholesterolemia from  electronic health records: The SEARCH study. J Clin Lipidol. 2016;10:1230-1239. |
| 13. Evidence   from Use |  |

CITATION: Electronic Health Record-based Phenotyping Algorithm for Familial Hypercholesterolemia [PseudoCode], version 2.0. Contributors: Iftikhar Kullo [Principal Investigator, Author], Adelaide Arruda-Olson, Carin Smith, Hongfang Liu, Majid Rastegar, Maya Safarova, Parvathi Balachandran, Saeed Mehrabi, Sunghwan Sohn, Xiao Fan, Yijing Cheng [Authors]. In: Phenotype Knowledgebase (PheKB). Created June 2016. Accessed May 12, 2020. Available at: https://phekb.org/sites/phenotype/files/FH_eAlgorithm_Pseudocode_FullText_2016_1_3.pdf.

COMMENTS:

Credit for metadata table: Flynn

**CBK Example I – Ontology**

| METADATA  CATEGORY | EXISTING METADATA |
| --- | --- |
| 1. Type | + is an ontology |
| 2. Domain | + is about antibiotics  + is about drugs  + is about medications  + is about antibiotic resistance |
| 3. Purpose | + is intended to describe antibiotic resistance genes and mutations, their products, mechanisms, and  associated phenotypes, as well as antibiotics and their molecular targets. |
| 4. Identification | + has title Antibiotic Resistance Ontology  + has short title ARO  + has version 1.0 |
| 5. Location | + has location <https://github.com/arpcard/aro>  + has location <https://raw.githubusercontent.com/arpcard/aro/master/aro.owl> |
| 6. CBK-to-CBK   Relationships | + is related to Comprehensive Antibiotic Resistance Database (CARD) |
| 7. Technical | + encoded in Web Ontology Language (OWL)  + has file type .owl  + has corresponding web page at <http://www.obofoundry.org/ontology/aro.html> |
| 8. Authorization & Rights Management | + has license Creative Commons CC-BY Version 4.0  + limitation of liability Under no circumstances, including but not limited to negligence, shall McMaster University be liable for any direct, indirect, special, punitive, incidental or consequential damages arising out of the use of, or the inability to use, the website or the Materials. |
| 9. Preservation |  |
| 10. Integrity |  |
| 11. Provenance | + last updated August, 2020  + owned by McMaster University, Ontario, Canada |
| TWO EVIDENCE CATEGORIES | |
| 12. Evidential   Basis |  |
| 13. Evidence   from Use |  |

CITATION: Antibiotic Resistance Ontology (ARO) [Terminology], version 1.0. In: OBO Library, entry aro. Revised August 2020. Accessed December 6, 2020. Available at: https://github.com/arpcard/aro. Computable resource at: https://raw.githubusercontent.com/arpcard/aro/master/aro.owl.

COMMENTS:

Credit for metadata table: Flynn

**CBK Example J – Order Set**

| METADATA  CATEGORY | EXISTING METADATA |
| --- | --- |
| 1. Type | + is an order set |
| 2. Domain | + is about clinical care  + is about endocrinology  + is about hypoglycemia  + is about hypoglycemia treatment |
| 3. Purpose | + is intended to guide treatment of hypoglycemia  + is intended to guide treatment for occurrences of hypoglycemic events  + intended for use in an adult population with an active problem of either diabetes mellitus, type 1 or  type 2  + Intended for use by clinical providers for Care and Management of Diabetic Patients |
| 4. Identification | + has title Endocrinology: Hypoglycemia Order Set  + has unique identifier b5c97034-fb05-5d16-9102-ca6b75b56d25  + has version 1.0 |
| 5. Location | + has location <https://cds.ahrq.gov/cdsconnect/artifact/endocrinology-hypoglycemia-order-set>  + has location AHRQ CDS Connect |
| 6. CBK-to-CBK   Relationships | + related to <https://cds.ahrq.gov/cdsconnect/artifact/endocrinology-hypoglycemia-rule> |
| 7. Technical | + has file size 8.6 Kb  + has file format XML |
| 8. Authorization & Rights Management | + copyright is held by Veterans Health Administration, Department of Veterans Affairs |
| 9. Preservation |  |
| 10. Integrity |  |
| 11. Provenance | + has status Draft  + has steward Veterans Health Administration  + published by Veterans Health Administration  + has contributors Leonard Pogach, MD, Paul Conlin, MD  + created on April 20, 2018  + publication date March 25, 2019  + last review date March 25, 2019 |
| TWO EVIDENCE CATEGORIES | |
| 12. Evidential   Basis | + based on <http://care.diabetesjournals.org/content/diacare/suppl/2016/12/15/40.Supplement_1.DC1/DC_40_S1_final.pdf> |
| 13. Evidence   from Use | + Not tested in a clinical environment |

CITATION: Endocrinology: Hypoglycemia Order Set [Clinical Decision Support Artifact], version 1.0. Contributors: Leonard Pogach, Paul Conlin [Contributors], Veterans Health Administration [Steward]. In: CDS Connect. Created April 20, 2018. Approved March 25, 2019. Accessed May 12, 2020. Available at: https://cds.ahrq.gov/cdsconnect/artifact/endocrinology-hypoglycemia-order-set.

COMMENTS:

Credit for metadata table: Flynn

**CBK Example K – Citation Resource**

| METADATA  CATEGORY | EXISTING METADATA |
| --- | --- |
| 1. Type | + is a citation resource |
| 2. Domain | + is about COVID-19  + is about remdesivir  + is about a FHIR resource of type Evidence |
| 3. Purpose | + is intended to convey citation information for an Evidence Resource |
| 4. Identification | + has identifier 58  + has title Citation for FEvIR Evidence 55 |
| 5. Location | + has location https://fevir.net/resources/Citation/58  + has URL https://fevir.net/resources/Citation/58 |
| 6. CBK-to-CBK   Relationships | + is derived from [https://fevir.net/resources/Evidence/55](https://computablepublishing.us/fevir/resources/Evidence/RemdesivirExampleWithSOLIDARITY?version=3) (CBK Example L) |
| 7. Technical | + encoded as JSON object |
| 8. Authorization & Rights Management | + has copyright information (for abstract) <https://creativecommons.org/licenses/by-nc-sa/4.0/> |
| 9. Preservation |  |
| 10. Integrity |  |
| 11. Provenance | + has status Active  + has publisher Computable Publishing LLC  + has author Brian S. Alper |
| TWO EVIDENCE CATEGORIES | |
| 12. Evidential   Basis | + is derived from https://fevir.net/resources/Evidence/55 (CBK Example L) – Evidential Basis is same as CBK-to-CBK Relationship for a citation of a CBK |
| 13. Evidence   from Use |  |

CITATION: Citation for FEvIR Evidence 55 [FHIR Resource]. Contributors: Brian S Alper [Author].  In: Fast Evidence Interoperability Resources (FEvIR) Platform, entry 58. Created March 13, 2021. Accessed March 13, 2021. Computable resource at: https://fevir.net/resources/Citation/58.

COMMENTS: Citations are not typically cited as distinct targets.

Credit for metadata table: Alper, Flynn

**CBK Example L – Evidence Resource**

| METADATA  CATEGORY | EXISTING METADATA |
| --- | --- |
| 1. Type | + is an evidence resource |
| 2. Domain | + is about COVID-19  + is about remdesivir  + is about 14-day mortality  + is about synthesis type = summary data meta-analysis  + is about study type = randomized trial |
| 3. Purpose | + is intended to convey structured representation of evidence  + is intended to convey evidence of effect of “exposure=Remdesivir IV 200 mg once then 100 mg once daily for 9 days” compared to “referenceExposure=Placebo” on “measuredVariable=Mortality at 14 days” in “population=Adults with COVID-19 pneumonia admitted to hospital” |
| 4. Identification | + has identifier 143 (from https://gps.health/coka)  + has identifier 55 (from https://fevir.net)  + has id 55  + has version 4  + has title “14-day mortality remdesivir vs placebo meta-analysis (ACTT-1, Wang et al, WHO SOLIDARITY)” |
| 5. Location | + has location https://fevir.net/resources/Evidence/55  + has URL https://fevir.net/resources/Evidence/55 |
| 6. CBK-to-CBK   Relationships | + is cited as https://fevir.net/resources/Citation/58 (CBK Example K)  + is related to (as a supporting citation) <http://computablepublishing.us/fevir/resources/Citation/4932> |
| 7. Technical | + encoded as JSON object |
| 8. Authorization & Rights Management | + has copyright information <https://creativecommons.org/licenses/by-nc-sa/4.0/> |
| 9. Preservation |  |
| 10. Integrity |  |
| 11. Provenance | + has status Active  + has publisher Computable Publishing LLC  + has authors Brian S. Alper, Joanne Dehnbostel, Khalid Shahin  + has contributions specified by authors as noted in the linked Citation Resource (CBK Example K) |
| TWO EVIDENCE CATEGORIES | |
| 12. Evidential   Basis | + The related artifacts include 3 citations which represent the 3 original studies that are the basis for this meta-analysis. |
| 13. Evidence   from Use |  |

CITATION: 14-day mortality remdesivir vs placebo meta-analysis (ACTT-1, Wang et al, WHO SOLIDARITY) [FHIR Resource], version 4. Contributors: Brian S Alper, Joanne Dehnbostel, Khalid Shahin [Authors].  In: Fast Evidence Interoperability Resources (FEvIR) Platform, entry 55. Created December 17, 2020. Revised December 21, 2020. Accessed March 13, 2021. Computable resource at: <https://fevir.net/resources/Evidence/55>.

COMMENTS:

Credit for metadata table: Alper
